# Albumin Counteracts Immune-Suppressive Effects of Lipid Mediators in Patients With Advanced Liver Disease

**DOI:** 10.1016/j.cgh.2017.08.027

**Published:** 2018-05

**Authors:** Louise China, Alexander Maini, Simon S. Skene, Zainib Shabir, Yvonne Sylvestre, Romain A. Colas, Lucy Ly, Natalia Becares Salles, Vittorio Belloti, Jesmond Dalli, Derek W. Gilroy, Alastair O’Brien

**Affiliations:** ∗ Division of Medicine, University College London, London, United Kingdom; ‡ University of London Comprehensive Clinical Trials Unit, London, United Kingdom; § Barts and The London School of Medicine and Dentistry, Queen Mary University of London, London, United Kingdom

**Keywords:** ATTIRE Trial, Resolution Phase Lipid Mediators, Immune Regulation, ALD, ACLF, acute-on-chronic liver failure, AD, acute decompensation, CI, confidence interval, CRP, C-reactive protein, HAS, human albumin solution, IL, interleukin, LM, lipid mediator, LPS, lipopolysaccharide, MDM, monocyte-derived macrophages, PGE_2_, prostaglandin E_2_, SD, standard deviation, TNF, tumor necrosis factor, WCC, white cell count

## Abstract

**Background & Aims:**

Patients with acute decompensation and acute-on-chronic liver failure (AD/ACLF) have immune dysfunction, which increases their risk for infections; however, there are no effective treatments to restore their immune function. We investigated whether the potentially immune-restorative effects of albumin are mediated by its effects on prostaglandin E_2_ (PGE_2_) and other lipids.

**Methods:**

We analyzed bloods samples from 45 of 79 patients with AD/ACLF and serum levels of albumin less than 30 g/L for whom infusion of 20% human albumin solution (HAS) increased serum levels of albumin 30 g/L or more in a feasibility study of effects of 20% HAS. Immune function was determined by comparison of macrophage function following addition of plasma samples. We also used samples from 12 healthy individuals. We measured binding of plasma proteins to PGE_2_ and serum levels of endotoxin (lipopolysaccharide) and cytokines; using 10 patients’ samples, we investigated the effects of PGE_2_ inhibitors. We performed a comprehensive lipid metabolomic analysis using samples from 10 different patients, before and after HAS administration.

**Results:**

At baseline, AD/ACLF patient plasma induced significantly lower production of tumor necrosis factor by healthy macrophages than plasma from healthy individuals (*P* < .0001). Plasma from patients after HAS infusion induced significantly higher levels of tumor necrosis factor production by macrophages (19.5 ± 4.8 ng/mL) compared with plasma collected before treatment (17.7 ± 4.5 ng/mL; *P* = .0013). There was a significantly lower proportion of plasma protein (albumin) binding to PGE_2_ from patients with AD/ACLF plasma (mean, 61.9%) compared with plasma from control subjects (77.1%; *P* = .0012). AD/ACLF plasma protein binding to PGE_2_ increased following HAS treatment compared with baseline (mean increase, 8.7%; *P* < .0001). Circulating levels of PGE_2_, lipopolysaccharide, and inflammatory or anti-inflammatory cytokines were higher in patients with AD/ACLF than healthy volunteers. Unexpectedly, HAS infusion had no effect on mediator levels. Principal component analysis of baseline levels of lipids that induce or resolve inflammation identified 2 distinct groups of patients that differed according to baseline plasma level of lipopolysaccharide. Sample analyses after HAS treatment indicated that albumin regulates circulating levels of lipid mediators, but this effect was distinct in each group.

**Conclusions:**

Analysis of blood samples from patients with AD/ACLF participating in a feasibility study of 20% HAS infusions has shown that infusions to raise serum albumin above 30 g/L reversed plasma-mediated immune dysfunction by binding and inactivating PGE_2_. We also describe a method to classify the inflammatory response in AD/ACLF, based on lipid profile, which could improve identification of patients most likely to respond to HAS treatment. A randomized controlled trial is needed to determine whether these effects of HAS reduce infections in AD/ACLF. Trial registered with European Medicines Agency (EudraCT 2014-002300-24) and adopted by NIHR (ISRCTN14174793).

See editorial on page 633, and related article on page 748.

A defective immune response in patients with acute decompensation (AD) or acute-on-chronic liver failure (ACLF) is widely considered to underlie susceptibility to bacterial infection.^1^, ^2^, ^3^ However, despite multiple studies the mechanisms underlying immune dysfunction in AD/ACLF remain unclear. We developed a model in which healthy volunteers’ monocyte-derived macrophages (MDMs) were incubated with plasma from patients with AD/ACLF and measured tumor necrosis factor (TNF) production, a validated marker of monocyte function in critical illness.^4^ Using this model, we demonstrated elevated plasma prostaglandin E_2_ (PGE_2_) and its potential role in immune suppression in patients with AD/ACLF. We also proposed a beneficial effect of transfusing 20% human albumin solution (HAS) to antagonize PGE_2_’s effects.^5^

Albumin has been reported to bind and catalyze PGE_2_ inactivation.^6^ Albumin is synthesized in the liver, therefore levels decrease in AD/ACLF, and so PGE_2_ should be more bioavailable. Defective functional binding capacity of albumin has been described in cirrhosis,^7^ again theoretically further enhancing bioavailability of PGE_2_. However the actual PGE_2_-albumin binding relationship in liver disease has never been explored. Studies have shown other potential immunomodulatory roles for albumin^8^, ^9^ but these have used samples from single center observational cohorts.

We performed immune function analysis of patients with AD using samples collected from a feasibility trial in preparation of the ATTIRE trial (Albumin To prevenT Infection in chronic liveR failure). Our feasibility trial included 79 patients with AD/ACLF who received 20% HAS. An accompanying manuscript in this issue details the clinical outcomes of these patients. The current article provides mechanistic insights into the potential immune restorative effect of targeted 20% HAS infusions in AD/ACLF.

Specifically, we aimed to confirm that elevated circulating PGE_2_ levels contributed to immune suppression; examine whether exogenous albumin improved PGE_2_-albumin binding and/or increased catalysis; compare PGE_2_ binding in commercial albumin preparations; determine whether any improvement in immune dysfunction observed following 20% HAS infusion was via a PGE_2_ effect; and examine the potential interaction of infused albumin with other plasma lipids (including proresolving mediators, molecules with host protective actions^10^), proinflammatory and anti-inflammatory cytokines, and endotoxin. Finally, we correlated laboratory findings with patient clinical characteristics.

## Methods and Analysis

### Study Design and Patients

ATTIRE’s protocol paper was published^11^ and the full protocol is available online. Ethical approval was granted by London-Brent research ethics committee (ref:15/LO/0104). All authors had access to study data and reviewed and approved the final manuscript. Studies were performed as follows with laboratory researcher blinded to whether the sample was pre- or post-HAS infusion.

### Laboratory Outcomes

The key secondary endpoint for ATTIRE feasibility study was change in immune function determined by patient plasma-induced healthy volunteer MDM dysfunction, as measured by endotoxin-stimulated TNF production (lipopolysaccharide [LPS]; *Salmonella abortus equi* S-form [TLRgrade, Enzo Life Science], NY), for 4 hours in presence of 25% patient plasma pre- and post-HAS treatment. TNF was measured with enzyme-linked immunosorbent assay (R&D systems, MN) as previously.^5^ Plasma samples analyzed were from admission (pre-HAS infusion) compared with samples once serum albumin had reached ≥30 g/L. The same assay was repeated using a monocyte cell line (mono-mac 6) for validation. Experiments were in a single centralized laboratory. Laboratory and matching clinical data were exchanged simultaneously between researcher and statisticians at the Comprehensive Clinical Trials Unit at University College London (Supplementary Methods).

### Plasma Protein Binding Capacity

Paired plasma samples pretreatment/post-treatment with 20% HAS were obtained from 52 of 79 patients in the ATTIRE feasibility trial. In 45 of 52 patients, the post-treatment sample corresponded to restoration of serum albumin ≥30 g/L (the primary endpoint) on mean treatment day 3.29 (standard deviation [SD], 1.27). These patients had mean pretreatment serum albumin 23.98 g/L (range, 12–29 g/L). In the other 7 of 52 patients the post-treatment sample was when patient had reached highest serum albumin level, and a sample had been taken that day. Plasma PGE_2_ binding was assessed in these samples with healthy volunteer samples (n = 12) as comparator.

The amount of PGE_2_ bound by plasma was determined using equilibrium dialysis (Thermo Scientific Single-Use RED [rapid equilibrium dialysis] Plate, IL), which enabled quantification of bound versus free PGE_2_ via postdialysis sample scintillation counting (Supplementary Methods). The 20% HAS from commercial suppliers Zenalb (BPL Herts, UK), Albunorm (Octapharm, Manchester, UK), and Alburex (CSL Behring, West Sussex, UK) including 2 different batches of Zenalb and Alburex were assessed and compared with fatty acid free albumin from human serum (Sigma-Aldrich, UK). Albumin concentration was diluted to 20 g/L (300 μM) and checked using bromocresol green.

### Monocyte-Derived Macrophage Functional Studies

We selected (while blinded) aliquots from 10 sample pairs that had showed at least a 15% difference in MDM TNF production following HAS infusion. A total of 15% was considered representative because 20% HAS infusions produced a mean >14% increase in MDM TNF (see results section). Experiments were performed with healthy volunteer plasma as comparator. PGE_2_ receptor antagonists AH6809 50 μM (EP1-3 antagonist) and MF498 10 μM (EP4 antagonist) (ie, pan PGE_2_ receptor blockade) were added to samples before LPS stimulation and TNF measured. Samples from Days 4, 5, and 10 of HAS treatment were also used.

### Lipopolysaccharide Detection and Cytokine Measurement

LPS and proinflammatory and anti-inflammatory cytokines were assayed in the 45 paired patient plasma samples using HEK293 cells and BD Cytometric Bead Array Human Soluble Protein Kit (BD Biosciences, Oxford, UK) (Supplementary Methods).

### Lipid Mediator Metabolomic Data

Samples from 10 patients pre- and post-HAS infusion from a top recruiting sites were chosen for analysis in view of the complexity of processing required and need for standardized collection and storage (Supplementary Methods).

### Statistical Analysis

For plasma analysis (a-d) a paired Student *t* test compared pre- and post-HAS treatment groups (Prism 7, CA). For lipid metabololipidomic analyses, Simca 14.1 (Malmo, Sweden) was used as below.

## Results

### Recruitment and Baseline Characteristics

Baseline characteristics were as follows: mean age, 53.4 years; male, 66%; and alcohol primary cause of cirrhosis, 96% (Supplementary Table 1). Mean Model for End-Stage Liver Disease score was 20.9 (SD, 6.62); 17 of 79 patients had ≥1 extra hepatic organ dysfunction at baseline and 21 (27%) ACLF grade 1–3. Baseline albumin levels were <25 g/L in 67%.

### Plasma-Mediated Immune Dysfunction Pre– and Post–20% Human Albumin Solution Infusions

**Figure 1:**
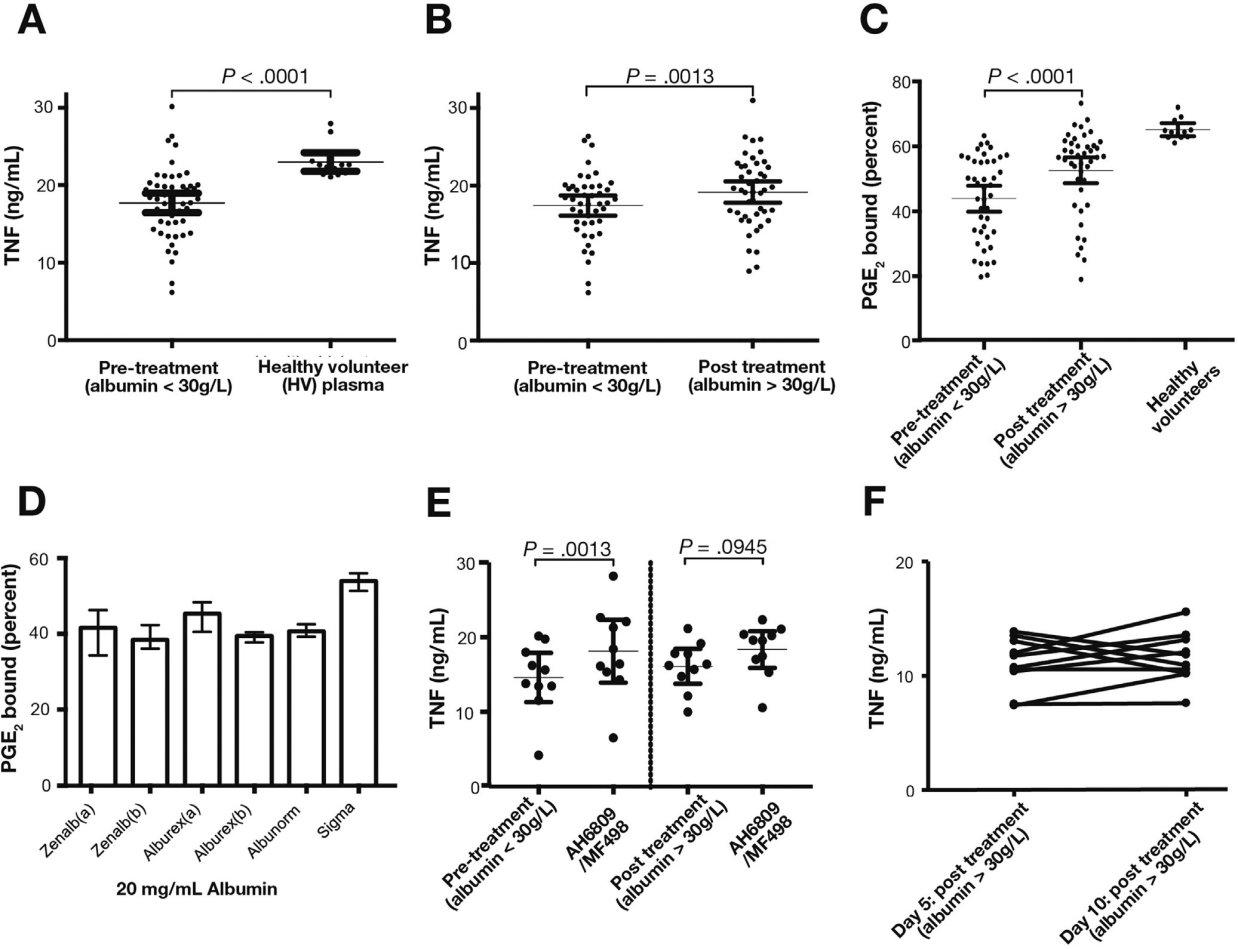
Targeted 20% HAS infusions reverse immune dysfunction in AD/ACLF by improving ability of patients with AD/ACLF plasma to bind PGE_2_. (*A*) Endotoxin (LPS) stimulated MDM TNF production in presence of patient plasma pretreatment with 20% HAS (n = 45 patients) compared with nonautologous healthy volunteer plasma. LPS MDMs TNF production in presence of healthy volunteer plasma was 6.88 ng/mL more in presence of AD plasma (CI, 4.85–8.91 ng/mL; *P* < .0001). (*B*) LPS MDM TNF production in presence of plasma pre- and post-HAS treatment (n = 45 patients incremented serum albumin >30 g/L). Mean post-treatment TNF increase 1.75 ng/mL (0.72–2.77; *P* = .0013), 14.5% (5.1%–23.5%). (*C*) Percentage PGE_2_/^3^H-PGE_2_ bound to healthy volunteer and AD/ACLF plasma protein using equilibrium dialysis. Post-HAS treatment plasma binds more PGE_2_ than pre-HAS (mean increase, 8.7%; CI, 5.2%–12.1%; *P* < .0001; n = 45). (*D*) Percentage PGE_2_/^3^H-PGE_2_ bound in different HAS products or Sigma albumin diluted to 20 g/L albumin in phosphate-buffered saline (n = 3). (*E*) LPS MDM TNF production in presence of pretreatment patient plasma (n = 10) in presence/absence of EP2 (AH6890-50 μM) and EP4 (MF498-10 μM) receptor antagonists compared with post-treatment effect. (*F*) LPS MDM TNF production in presence of AD/ACLF plasma Day 5 and 10 post-treatment with 20% HAS.

**Supplementary Figure 1:**
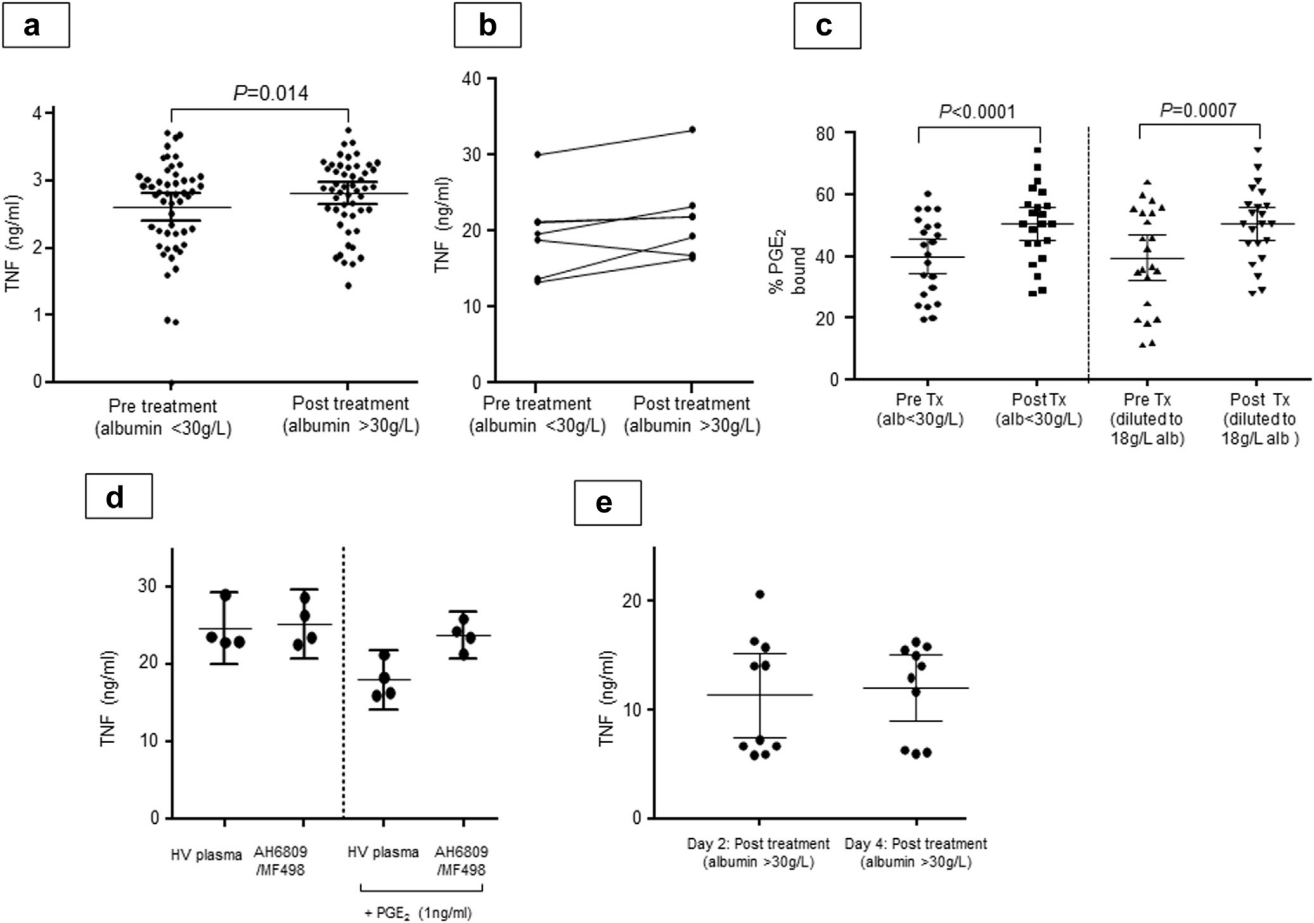
Targeted 20% HAS infusions reverse immune dysfunction in AD/ACLF by improving AD/ACLF plasma PGE_2_ binding. (*A*) TNF production from MonoMac-6 cell line (MM6) stimulated with LPS in the presence of patient plasma pretreatment and post-treatment with 20% HAS (n = 45 patients as above). Mean increase in MM6 TNF-α production was 0.211 ng/mL (10.2%; CI, 0.0517–0.369 ng/mL; *P* = .014). (*B*) TNF production from healthy volunteer MDMs stimulated with LPS in the presence of patient plasma pretreatment and post-treatment with 20% HAS (n = 7 patients; patients that did not increment serum albumin to ≥30 g/L and had a pretreatment and post-treatment sample available for analysis). Sample numbers too small for statistical analysis. (*C*) Percentage of PGE_2_/^3^H-PGE_2_ bound to patient plasma protein using equilibrium dialysis, comparing patient plasma pretreatment and post-treatment with 20% HAS. Data shown with undiluted samples and when all samples had been diluted to the same albumin concentration (18 g/L). (*D*) TNF production from healthy volunteer MDMs stimulated with LPS in presence of healthy plasma (n = 4) and presence/absence of AH6890 (50 μM) and MF498 (10 μM) and 1 ng/mL PGE_2_. (*E*) TNF production from healthy volunteer MDMs stimulated with LPS in the presence of patient plasma post-treatment with 20% HAS, samples Day 2 and 4 post-treatment. HV, healthy volunteer; Tx, treatment.

Patient plasma treatment significantly reduced endotoxin (LPS)-stimulated production of TNF from healthy MDMs compared with healthy volunteer plasma (*P* < .0001) (Figure 1*A*). There was a significant increase in MDM TNF production of 14.3% (95% confidence interval [CI], 5.1%–23.5%; 17.7 ± 4.5 ng/mL to 19.5 ± 4.8 ng/mL; *P* = .0013) (Figure 1*B*) following addition of post-HAS treatment plasma compared with pretreatment from 45 paired samples. In total, 30 of 45 (78%) had improved MDM TNF production post-treatment. A differentiated monocyte cell line showed similar findings of 10.2% (95% CI, 2.5%–17.9%; *P* = .014) (Supplementary Figure 1*A*). There was a trend toward increased TNF production from patients not incrementing ≥30 g/L (n = 7) compared with pretreatment (Supplementary Figure 1*B*). There was no change in mean white cell count (WCC) or C-reactive protein (CRP) between pre and post samples but serum bilirubin was reduced by a mean 25% (Supplementary Table 2).

### Targeted 20% Human Albumin Solution Infusions Improved Acute Decompensation and Acute-on-Chronic Liver Failure Plasma Ability to Bind Prostaglandin E_2_ Both by Increasing Albumin Concentration and Functional Capacity With No Effect on Overall Prostaglandin E_2_ Concentration

Plasma PGE_2_ concentrations pretreatment were highly variable but substantially elevated with a mean 52.5 pg/mL (SD, 44.6; n = 10) compared with published healthy volunteer concentrations using this technique (mean, 4.1; SD, 0.2 pg/mL).^12^ Albumin infusion had no overall effect on total plasma PGE_2_ concentrations, which measure free and albumin-bound PGE_2_ (pretreatment, 52.5 [13.4] pg/mL vs post-treatment, 49.9 [8.1] pg/mL) (Supplementary Table 3, Supplementary Table 4).

Albumin bound PGE_2_ with very low affinity and calculated dissociation constant was approximately 280 μM. This low binding affinity suggests that decreasing serum albumin to AD/ACLF patient levels combined with observed increases in PGE_2_ concentration could result in increases in free circulating PGE_2_ to pathophysiological levels. To illustrate this we performed theoretical calculations (Supplementary Table 5).

AD/ACLF plasma bound a mean of 15.2% less PGE_2_ compared with healthy volunteer plasma (n = 12; 77.1% vs 61.9%; *P* = .0012). The 45 paired patient samples showed an improved ability to bind PGE_2_ post-HAS treatment with mean increase of 8.7% (95% CI, 5.2%–12.1%; *P* < .0001) (Figure 1*C*).

The binding may have improved because of increased plasma albumin concentration following treatment. To investigate functional alterations in binding we selected 23 patient samples with a greater or equal improvement in binding compared with overall mean 8.7% PGE_2_ bound (mean, 16.1%; 95% CI, 6.0%–15.0%; *P* < .0001). Pretreatment and post-treatment plasma was diluted to 18 g/L albumin and post-treatment plasma bound significantly more PGE_2_ than pretreatment (mean increase, 10.9%; 95% CI, 5.2%–16.7%; *P* = .0007) (Supplementary Figure 1*C*) suggesting functional improvement in binding capacity.

### Commercially Available 20% Human Albumin Solution Tested Bound Prostaglandin E_2_ to a Similar Degree

There were no significant differences in PGE_2_ binding among samples tested (Figure 1*D*) and values were less than healthy volunteer plasma binding.

### 20% Human Albumin Solution Infusions Seem to Improve Immune Function in Patients With Acute Decompensation and Acute-on-Chronic Liver Failure by Reversing the Immune Suppressive Effect of Prostaglandin E_2_ With Effect Maintained to at Least Day 10 of Treatment

LPS-induced TNF production from MDMs pretreated with pan-PGE_2_ receptor blockade (EP1-3-AH6890 and EP4-MF698) before addition of pre-HAS treatment plasma was increased to a similar level as when post-HAS plasma was added (without PGE_2_ antagonists). Mean increase was 3.51 ng/mL (*P* = .0013; 95% CI, 1.78–5.24) (Figure 1*E*). However pan-PGE_2_ receptor blockade had no significant effect on MDMs treated with post–20% HAS plasma (*P* = .0945). These antagonists had no effect on MDM TNF production when added to healthy plasma samples (Supplementary Figure 1*D*). The increased MDM TNF production between pre- and post-HAS treatment was maintained but not increased up to Day 10 of treatment in 10 samples analyzed (Figure 1*F*, Supplementary Figure 1*E*).

### Targeted 20% Human Albumin Solution Infusions had No Significant Effect on Elevated Plasma Concentrations of Lipopolysaccharide and Proinflammatory/Anti-Inflammatory Cytokines Seen in Patients With Acute Decompensation and Acute-on-Chronic Liver Failure

There was a trend toward reduction but no significant differences in total plasma proinflammatory and anti-inflammatory cytokine levels assayed (TNF, interleukin [IL] 1β, IL6, IL10, and IL8) and LPS concentrations in 45 paired samples (Supplementary Table 6).

### Principal Component Analysis of Baseline (Pretreatment) Inflammation Initiating and Proresolving Plasma Lipid Mediators Identified 2 Distinct Acute Decompensation and Acute-on-Chronic Liver Failure Patient Groups and Targeted 20% Human Albumin Solution Infusions Demonstrated Distinct Responses Between These Groups

**Figure 2:**
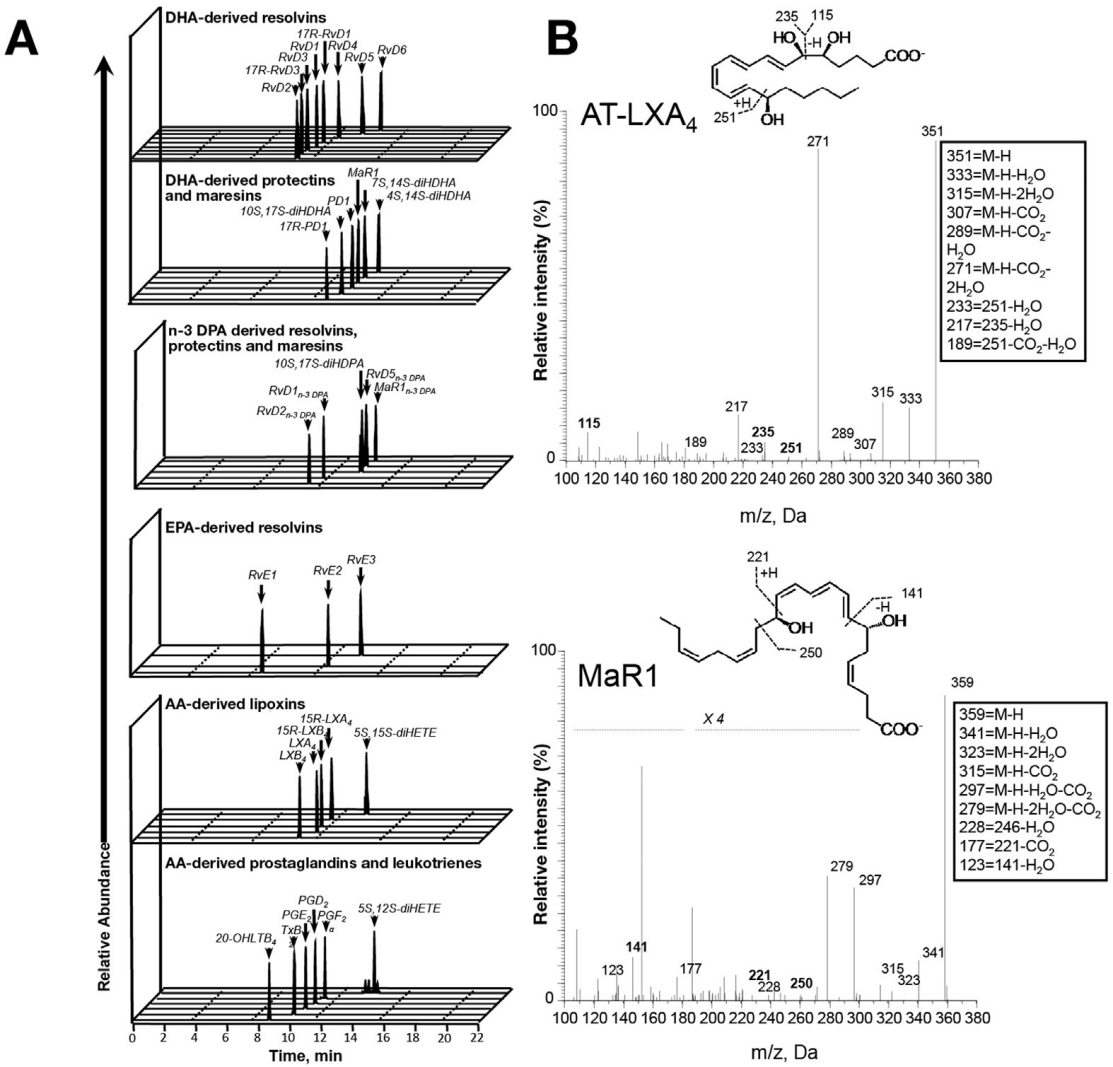
AD/ACLF plasma proresolving and inflammation-initiating mediator profiles. Plasma collected pre and post albumin administration and lipid mediators profiled using liquid chromatography–tandem mass spectrometry LM metabololipidomics. (*A*) Representative multiple reaction chromatograms for identified lipid mediators and (*B*) tandem mass spectrometry spectrum used in identification of AT-lipoxins A_4_ and MaR1. Representative of 10 patients. AA, arachidonic acid; DHA, docosahexaenoic acid; DPA, docosapentaenoic acid; EPA, eicosapentaenoic acid; m/z, mass to charge ratio.

We investigated plasma lipid mediator (LM) profiles for essential fatty acid–derived (docosahexaenoic acid, n-3 docosapentaenoid acid, eicosapentaenoic acid, and arachidonic acid), proresolving mediators: resolvins, protectins, maresins, and lipoxins in 10 plasma samples pre–20% HAS infusion and once serum albumin had reached 30 g/L following treatment. We quantified the classic inflammation-initiating eicosanoids (prostaglandins, thromboxane B_2_, and leukotrienes). Identification was conducted in accordance with published criteria including matching retention time and at least 6 diagnostic ions in tandem mass spectrum^12^ (Figure 2, Supplementary Table 3, Supplementary Table 4).

**Figure 3:**
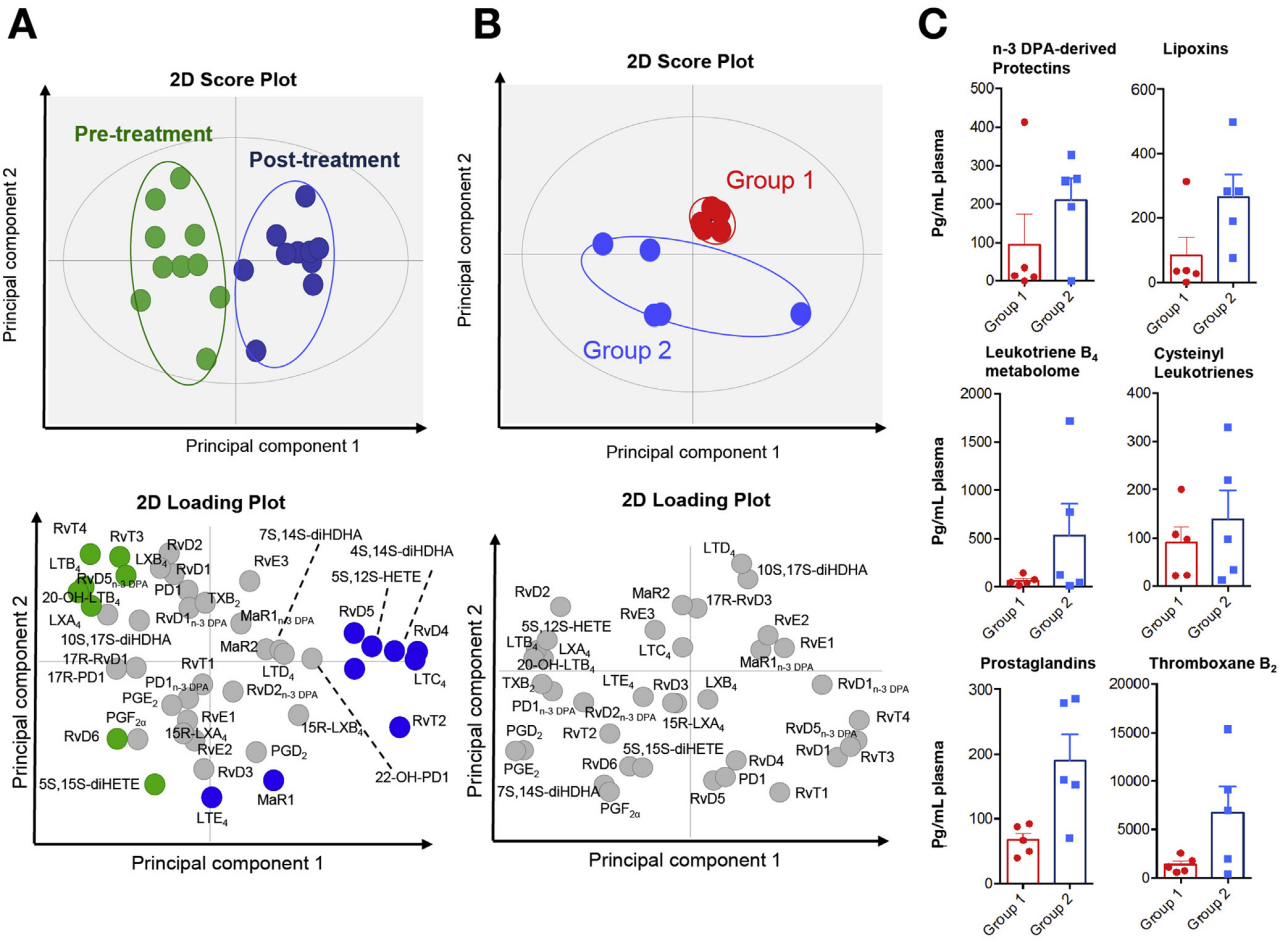
Albumin administration shifts AD/ACLF plasma LM profiles and prealbumin LM profiles identifies 2 distinct AD/ACLF patient groups with a hyperactivated (group 2) and hypoactivated (group 1) LM phenotype. LM profiles interrogated using partial least square discriminant analysis. (*A*) 2D score plot with baseline and post treatment plasma LM profiles interrogated using principle component analysis. 2D score plot (top), 2D loading plot (*bottom*). (*B*) 2D loading plot with pre-treatment plasma pro-resolving and inflammation-initiating mediators identifies 2 distinct groups: group 1 (*red*) and group 2 (*blue*). (*C*) Assessment of lipid mediator profiles in each of these groups pre albumin treatment demonstrated a hypo-activated profile (Group 1) with reduced concentrations of several proresolving and inflammation initiating mediators. The second hyper-activated group was characterized by overall elevated lipid mediator concentrations (Group 2). Results mean ± standard error of the mean, representative of 10 patients.

We identified mediators from each major essential fatty acid metabolome including 13 series resolvins 1, protectins 1, and lipoxins A_4_. Using multivariate analysis of plasma LM profiles pre and post albumin treatment we found that each of these groups segregated into 2 distinct clusters (Figure 3*A* and *B*). These data indicate that albumin treatment regulates circulating LM levels. However, overall LM levels pre and post albumin treatment did not demonstrate statistically significant changes (Supplementary Table 3, Supplementary Table 4). Therefore, we questioned whether responses in plasma LM profiles following albumin were dependent on pretreatment LM levels.

**Figure 4:**
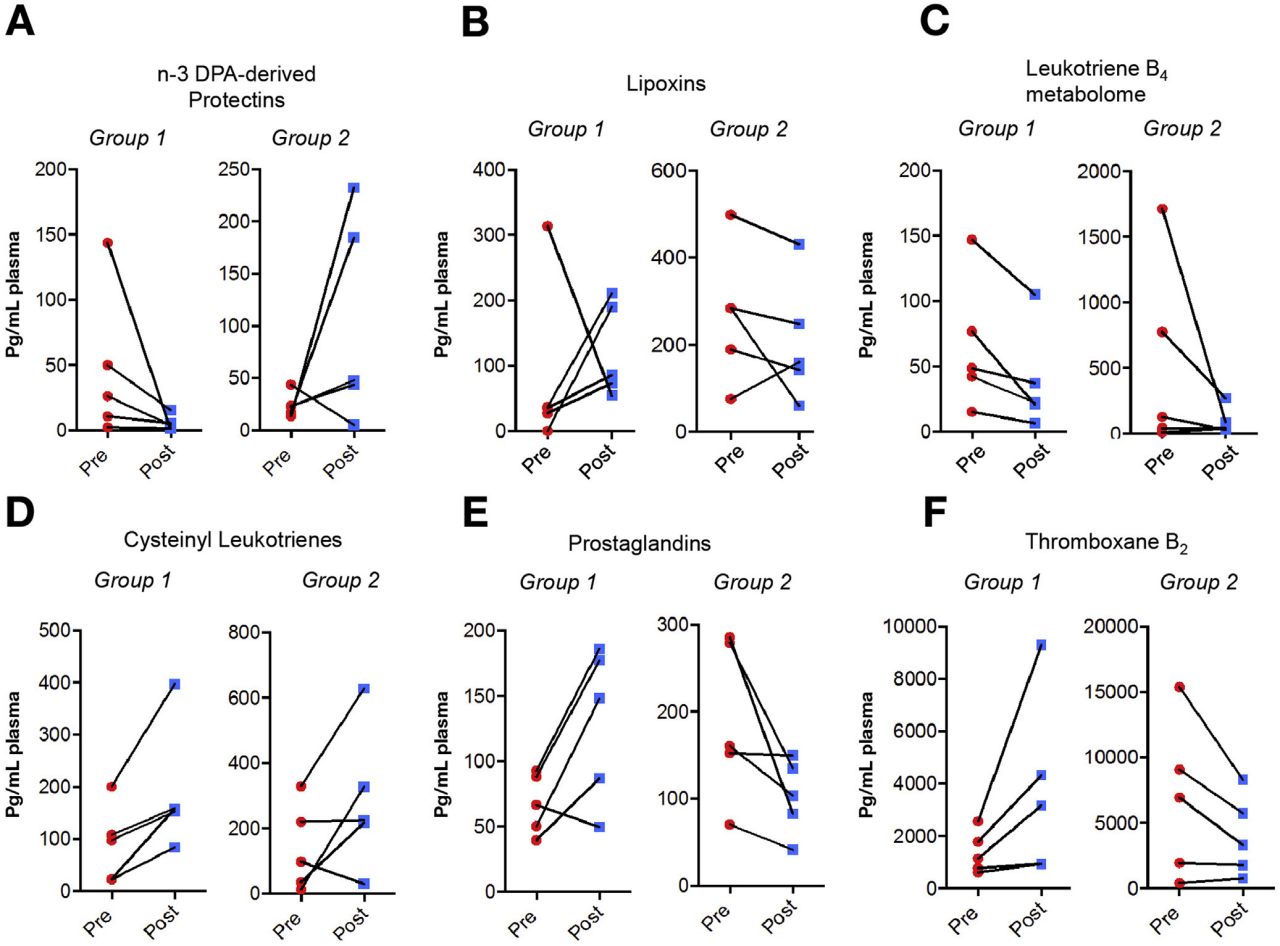
Albumin differentially regulates plasma LM profiles in patients with AD/ACLF with hyperactivated and hypoactivated LM phenotypes. Plasma pre and post albumin administration was LM profiled using liquid chromatography–tandem mass spectrometry based LM metabololipidomics and quantified using multiple reaction monitoring. (*A–F*) Results represent cumulative levels of proresolving mediator and inflammation-initiating eicosanoids found to be regulated by albumin administration. Five patients per group per interval.

Principal component analysis of pretreatment LM profiles identified 2 distinct patient groups characterized by 5 patients per group (Figure 3*B*). Assessment of LM profiles in each group pre albumin treatment demonstrated a hypoactivated profile (Group 1) with reduced concentrations of several proresolving and inflammation-initiating mediators including n-3 docosapentaenoic acid–derived protectins, AA-derived lipoxins and prostaglandins, thromboxane B_2_, and leukotriene B_4_ (Figure 3*C*). The second hyperactivated group demonstrated overall elevated LM concentrations (Group 2; Figure 3*B* and *C*). Patients in the hyperactivated group had elevated WCC, temperature, cytokine, and CRP levels and statistically significant increases in plasma endotoxin concentration (Table 1). Investigation of peripheral blood LM levels pre and post albumin administration demonstrated a re-equilibration of several mediator families. LM concentrations for several of the families identified in the hyperactivated group were found to be decreased, whereas in the hypoactivated group mediator concentrations increased post albumin treatment (Figure 4*A–F*). These results demonstrate that plasma LM profiles identify 2 distinct patients groups, hypoactivated and hyperactivated, and regulation of plasma LM profiles by albumin is distinct in each. The endotoxin and cytokine levels did not change significantly following HAS in either group and there was no difference in clinical outcomes.

**Table 1:** Clinical Characteristics of Group 1 (Hypoinflammatory) and Group 2 (Hyperinflammatory) Patients as Defined by Lipid Mediator Principal Component Analysis at Baseline (n = 5 Patients per Group)

	Group 1: hypoinflammatory lipid mediator profile, mean (SD)		Group 2: hyperinflammatory lipid mediator profile, mean (SD)	
MELD	18.9 (5.4)		20.1 (7.9)	
Age	45.66 (13.52)		48.63 (13.85)	
	Pre 20% HAS	Post 20% HAS	Pre 20% HAS	Post 20% HAS
Serum albumin (g/L)	22.4 (6.1)	28.2 (5.2)	20.8 (3)	29.6 (4.5)
Temperature (°C)	36.84 (1.0)		37.96 (1.1)	
White cell count (x10^9^/L)	11.24 (4.5)	9.18 (5.83)	18.16 (14.2)	4.6 (16.4)
CRP (mg/mL)	70.8 (76)	107.0 (46.8)	92.6 (93.3)	46.0 (45.4)
Heart rate (bpm)	104.4 (14.5)	Not available	106.8 (23.7)	Not available
Endotoxin (pg/mL)	3.7 (2.3)	7.2 (3.5)	23.44 (12.3)	19.0 (13.3)
TNF (pg/mL)	0.75 (0.5)	3.0 (1.7)	2.27 (1.7)	5.4 (7.5)
IL1b (pg/mL)	0.67 (1.5)	1.6 (1.7)	4.13 (6.8)	2.3 (2.8)
IL6 (pg/mL)	227.7 (407.2)	7135 (6649)	898.1 (1949.4)	14,121 (31,356)
IL8 (pg/mL)	372.9 (96.9)	576.7 (285.1)	442.3 (173.2)	828.2 (1271.9)
IL10 (pg/mL)	0.6 (3.2)	120.0 (110.5)	12.9 (12.6)	239.9 (13.3)

CRP, C-reactive protein; HAS, human albumin solution; IL, interleukin; MELD, model for end-stage liver disease; SD, standard deviation; TNF, tumor necrosis factor.

## Discussion

This represents the first demonstration of a potential pharmacological immune restorative role for 20% HAS infusions in patients with AD/ACLF through its ability to bind PGE_2_ using samples from a multicenter interventional trial. We show that 20% HAS infusions seemed to reverse AD/ACLF patient plasma-induced macrophage dysfunction restoring TNF production towards levels seen when macrophages were incubated with healthy plasma. We had no control arm and this may be caused by other patient care aspects because median time between samples was 4 days, with an overall 25% improvement in bilirubin observed, which cannot be excluded as a confounder. We again show significantly elevated prostaglandins and demonstrate for the first time proresolving mediators resolvins, protectins, maresins, and lipoxins in patients with AD/ACLF. These autacoids stimulate key cellular resolution events, enhancing macrophage apoptotic cell clearance.^10^ Finally principal component analysis of LMs divided patients into a hyperinflammatory and hypoinflammatory profile that could be differentiated by plasma endotoxin concentrations from 10 patients analyzed with nonsignificantly elevated WCC, temperature, cytokine, and CRP levels. Importantly, 20% HAS infusions seemed to equilibrate the inflammatory balance of inflammation initiating eicosanoids and proresolving mediators between these groups without affecting endotoxin or cytokine levels. These data suggest a further novel immune restorative effect for albumin.

Albumin is considered to have immune modulatory effects in AD/ACLF^13^, ^14^, ^15^ but no prospective trial has identified mechanistic action beyond volume expansion. We previously demonstrated its potential to antagonize the effects of PGE_2_ and others have suggested it binds endotoxin or exerts a beneficial effect on proinflammatory cytokines.^16^, ^17^, ^18^ Immune function is an extremely complex process and we designed a pragmatic assay to investigate samples from multiple sites that we have validated by showing similar effects in freshly isolated monocytes from patients with ACLF.^19^ We show for the first time in a prospective trial that 20% HAS infusion reversed the immune suppressive effects of PGE_2_ in AD/ACLF by improving plasma binding to this molecule, thereby inactivating it. This effect persisted in samples tested to Day 10 but did not improve once serum albumin was >30 g/L. PGE_2_ binds albumin but no other plasma proteins^20^ and we found albumin to have a very low binding affinity for PGE_2_ supporting the presence of free unbound PGE_2_ within plasma at pathophysiological concentrations. Albumin infusion improved plasma protein binding to PGE_2_. Studies using PGE_2_ (E-prostanoid) receptor antagonists demonstrated a similar immune restorative effect to 20% HAS infusion and had no effect in samples post-HAS infusion supporting this immune restorative effect of albumin occurring via PGE_2_ inhibition. Unexpectedly plasma endotoxin and proinflammatory/anti-inflammatory cytokine concentrations were unaffected by albumin infusions, therefore effects observed were not via direct modulation of these. We found no difference in the ability of commercial albumins tested to bind PGE_2._ Patients recruited to our randomized controlled trial will be given HAS customarily used at that site and therefore the absence of any differences between manufacturers was important.

Overall PGE_2_ concentrations were unaffected by HAS infusion, which challenges previous data demonstrating PGE_2_ catalysis by albumin.^6^ This observation may be related to our assay measuring total PGE_2_, both free and albumin bound. We hope to develop techniques to differentiate between the 2 to determine whether free PGE_2_ is catalyzed by albumin.

Albumin is present in low concentrations in AD/ACLF and has decreased functional efficacy^7^ caused by post-transcriptional modification.^21^ Administration of 20% HAS not only improved albumin concentration but also functional capacity to bind immunosuppressive PGE_2_. Taken together these data suggest that 20% HAS infusions act pharmacologically to improve immune function in AD/ACLF through albumin’s ability to bind elevated circulating levels of immunosuppressive PGE_2_. Its weak binding of PGE_2_ and lack of effect on absolute levels may explain the absence of renal or gastrointestinal side effects in contrast to nonsteroidal anti-inflammatory drugs, which alter eicosanoid profiles at these sites. Studies have identified structural and functional alterations in commercial HAS compared with healthy albumin^22^ and we showed reduced PGE_2_ binding compared with healthy volunteer plasma. It may be the immune effects of albumin could be enhanced with further research. Again we observed heterogeneity in immune dysfunction and response to albumin using our immune assay may identify patients most likely to benefit from this approach.

A weakness was the lack of a control (untreated arm) and therefore immune function may have improved because of patients recovering; indeed serum bilirubin fell by 25% between samples. However the CRP and WCC were unchanged, and we previously showed immune dysfunction in AD persisted throughout hospital admission.^10^ Furthermore, plasma cytokine and endotoxin levels did not alter between pre- and post-HAS samples, which might have been expected to fall if the patients were substantially better.

**Figure 5:**
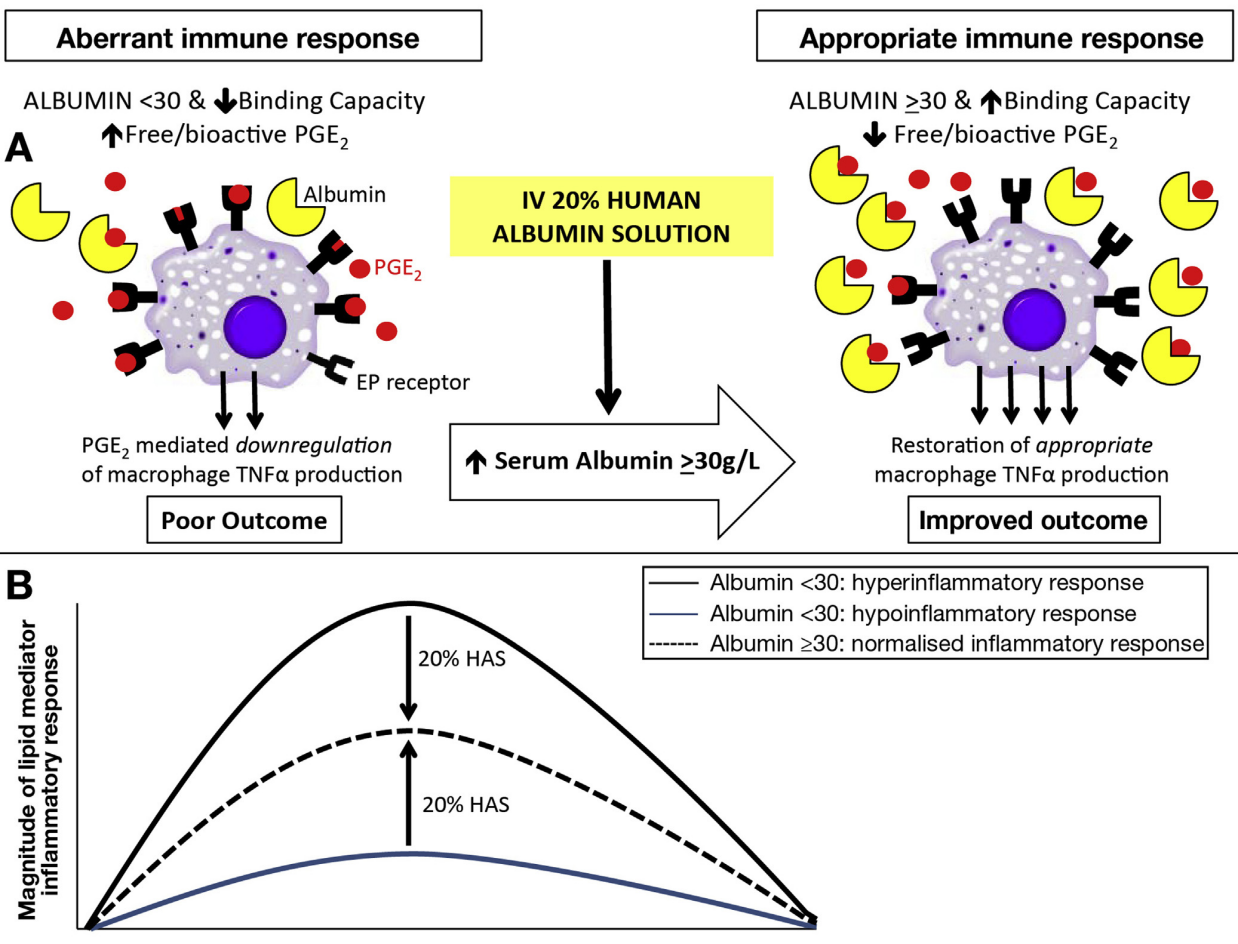
Schematic version of our hypothesis that prophylactic human albumin infusions improve immune response in ACLF via 2 mechanisms. (*A*) HAS increases circulating albumin concentration and function improving binding of immunosuppressive PGE_2_, reducing free/bioactive PGE_2_ and restoring monocyte/macrophage function. (*B*) Patients with ACLF can be divided into hypoinflammatory and hyperinflammatory responses defined by LM metabolomics, both with potential adverse outcomes. HAS rectifies this LM imbalance leading to a normalized, appropriate inflammatory response with potential improved outcome. IV, intravenous

Plasma LM profiling of the 4 major essential fatty acid bioactive metabolomes demonstrated albumin administration caused a shift in peripheral blood LM profiles. Post hoc analysis of prealbumin LM profiles identified 2 distinct groups, a hyperactivated profile with elevated levels of inflammation-initiating eicosanoids and proresolving mediators and a hypoactivated profile with reduced LMs. Albumin administration led to distinct regulation of LM profiles in each group suggesting that it may activate different protective mechanisms in these groups. Immunophenotyping sepsis studies have shown both hyperactivated and hypoactivated profiles can lead to a negative outcome^23^, ^24^; indeed recent evidence has shown that a hyperactivated plasma lipid signature predicts death in sepsis.^25^ Albumin may therefore have further beneficial immune effects. The potential role of these LMs in inflammation and infection and possible utility of LM immunophenotyping has never previously been described in liver disease. Although the hyperactivated group had elevated concentrations of endotoxin and cytokine pretreatment, these levels were unaffected by HAS infusion. These data therefore offer a completely novel opportunity to study the effect of albumin on the immune system. Figure 5 provides a schematic version of our hypothesis.

In summary using samples from our multicenter feasibility study we present novel evidence that targeted albumin infusions seem to exert a beneficial immune effect in patients with AD/ACLF via its ability to bind PGE_2_, but do not reduce overall total circulating levels. We identify for the first time proresolving LMs in advanced liver disease and propose that LM metabolic analysis could immunophenotype these patients. Finally, a second novel potential immune restorative role in which albumin infusions rectify both hyperinflammatory and hypoinflammatory LM profiles was demonstrated. We believe our study provides the first evidence for an immune-based mechanism of 20% HAS in AD/ACLF. However, a control arm was not included in the study design. Sample collection from ATTIRE stage 2, our randomized controlled trial to assess whether our 20% HAS infusion regimen leads to a reduction in infection, renal dysfunction, and death in patients with AD/ACLF compared with standard care, will provide further opportunity to investigate the role of these LMs in AD/ACLF.

## Figures and Tables

**Supplementary Table 1:** Baseline Demographics and Clinical Characteristics of the Analysis Population (n = 79)

Characteristic	Mean (SD)
Age, *y*	53.41 (11.63)
Serum albumin, *g/L*	23.95 (3.51)
Days since admission	1.81 (0.88)
MELD	20.90 (6.62)
Creatinine	91.2 (78.2)
	n (*%*)
Male	52 (66)
Admitted to ICU	2 (3)
Prescribed antibiotics	41 (52)
Diagnosis of infection	27 (34)

Etiology of cirrhosis^a^	n (*%*)
Alcohol	76 (96)
Hepatitis B	1 (1)
Hepatitis C	11 (14)
NAFLD	4 (5)
Other etiologies	2 (3)

Organ failure according to proposed definitions	n (*%*)
Renal	8 (10)
Respiratory	9 (11)
Circulatory	13 (16)
Brain	3 (4)

ACLF grade^b^	n (*%*)
Grade 0	58 (73)
Grade 1	11 (14)
Grade 2	6 (8)
Grade 3	4 (5)

ACLF, acute-on-chronic liver failure; ICU, intensive care unit; MELD, Model for End-Stage Liver Disease; NAFLD, nonalcoholic fatty liver disease; SD, standard deviation.

a Some patients have more than 1 liver cirrhosis etiology.

b According to European foundation for the study of chronic liver failure criteria.

**Supplementary Table 2:** Differences in Blood Test Values Pre-HAS Infusion and After Albumin Restored ≥30 g/L in Samples Used for Immune Function Analysis

	Mean (SD) Pre–20% HAS infusion, serum albumin <30 g/L	Mean (SD) serum albumin restored ≥30 g/L	*P* value (paired Student *t* test)
White cell count	9.3 (6.4)	8.6 (7.5)	.23
CRP	44 (52)	30 (31)	.39
Bilirubin	166 (154)	145 (116)	.02

CRP, C-reactive protein; HAS, human albumin solution; SD, standard deviation.

**Supplementary Table 3:** Plasma LM Profiles in Prealbumin Administration LM Levels Were Assessed Using LM Metabololipidomics

DHA bioactive metabolome	Q1	Q3	Group 1					Group 2				
			2-2529 AOB	3-2445 AOB	8-1822 AOB	9-2417 AOB	10-2200 AOB	5-1026 AOB	4-1486 AOB	1-2320 AOB	6-2419 AOB	7-1702 AOB
RvD1	375	141	—	—	2.2	0.8	2.7	3.3	1.7	19.1	1.0	1.8
RvD2	375	141	2.9	1.7	2.1	0.9	3.8	—	—	—	4.9	11.2
RvD3	375	147	—	—	2.8	5.1	0.7	3.1	3.4	1.1	0.0	0.6
RvD4	375	101	—	—	0.2	0.7	0.8	1.0	0.5	1.5	1.3	0.2
RvD5	359	199	7.2	6.8	—	1.3	—	12.4	3.3	13.8	4.6	8.6
RvD6	359	101	—	—	0.8	—	0.6	23.1	115.0	—	5.0	—
17R-RvD1	375	141	139.6	0.2	—	—	—	—	—	—	—	—
17R-RvD3	375	147	—	—	15.1	0.3	—	—	—	—	—	2.6
PD1	359	153	0.3	3.8	—	—	—	—	10.7	7.4	3.3	1.0
17R-PD1	359	153	—	1.1	7.8	3.9	3.6	—	—	1.6	3.5	—
10S,17S-diHDHA	359	153	—	1.1	—	—	11.1	—	—	—	—	—
22-OH-PD1	375	153	1.7	—	—	—	—	0.9	—	—	—	—
MaR1	359	221	—	—	—	—	—	1.1	—	—	—	—
7S,14S-diHDHA	359	221	—	—	—	11.5	—	—	—	6.6	—	—
4S,14S-diHDHA	359	101	10.3	—	4.9	4.9	—	1.8	—	—	—	4.7
n-3 DPA bioactive metabolome												
RvT1	377	239	3.0	1.4	1.2	2.8	0.3	3.5	3.5	4.4	2.1	0.3
RvT2	377	197	0.6	—	—	—	—	1.4	—	—	0.3	0.3
RvT3	377	197	2.3	0.7	3.3	—	1.5	—	2.0	20.6	0.3	1.0
RvT4	377	211	9.5	3.0	33.3	—	8.4	—	7.9	77.7	1.5	5.1
RvD1_n-3 DPA_	377	143	47.4	2.3	12.7	5.0	—	2.9	3.7	35.5	—	6.8
RvD2_n-3 DPA_	377	215	2.2	2.2	—	0.8	—	1.7	2.2	—	—	2.3
RvD5_n-3 DPA_	361	199	—	—	26.0	—	—	9.5	—	266.9	3.4	—
PD1_n-3 DPA_	361	183	10.9	34.2	413.2	13.8	—	259.8	265.9	—	192.7	327.9
10S,17S-diHDPA	361	183	—	—	—	—	—	—	—	—	—	—
MaR1_n-3 DPA_	361	223	154.1	7.4	—	12.1	—	—	—	30.4	13.6	—
7S, 14S-diHDPA	361	223	—	8.3	—	6.2	—	15.0	41.4	18.4	5.5	28.7
EPA bioactive metabolome												
RvE1	349	195	1.6	—	1.1	—	20.7	1.5	0.8	7.6	—	0.6
RvE2	333	199	6.6	—	11.1	—	118.1	9.1	3.2	29.1	—	3.0
RvE3	333	201	2.5	26.2	37.9	2.4	5.0	3.1	18.8	7.1	23.7	13.8
AA bioactive metabolome												
LXA_4_	351	217	0.8	—	—	—	1.0	—	1.2	—	1.7	8.0
LXB_4_	351	221	16.0	—	147.4	—	—	—	64.0	169.6	44.0	167.8
5S,15S-diHETE	335	235	10.8	—	148.3	35.0	29.4	486.1	118.4	110.1	29.6	83.1
AT-LXA_4_	351	217	—	—	17.7	—	—	12.0	6.1	3.5	—	3.5
AT-LXB_4_	351	221	—	—	—	—	6.6	—	—	—	—	21.9
LTB_4_	335	195	40.6	33.4	9.1	15.9	93.3	27.5	40.2	7.0	281.3	596.6
5S,12S-diHETE	335	195	4.5	0.4	2.4	11.1	3.3	5.3	2.4	1.3	9.7	13.0
20-OH-LTB_4_	351	195	3.6	43.0	3.8	15.3	50.4	11.6	87.1	4.0	486.7	1106.0
LTC_4_	626	189	12.2	35.8	38.7	139.5	28.9	88.6	—	11.7	—	23.0
LTD_4_	497	189	—	12.4	17.7	17.2	7.8	—	—	—	—	—
LTE_4_	440	189	0.8	59.3	144.1	62.9	61.1	240.2	34.9	10.7	97.9	0.1
PGD_2_	351	189	8.1	13.1	4.1	30.1	14.9	55.5	36.9	6.0	34.0	34.7
PGE_2_	351	189	14.0	23.4	32.4	11.4	24.8	111.9	105.2	7.5	112.5	81.5
PGF_2α_	353	193	25.5	26.9	60.4	55.1	63.2	167.5	180.4	62.8	48.2	71.2
TxB_2_	369	169	1142.4	623.8	2566.2	780.2	1781.9	1939.9	6946.8	408.4	15370.6	9075.9

NOTE. Results are expressed as pg/mL. Em dash = below limit; limit ≈ 0.1 pg. With Q1, M-H (parent ion); and Q3, diagnostic ion in the tandem mass spectrometry (daughter ion).

AA, arachidonic acid; DHA, docosahexaenoic acid; DPA, docosapentaenoic acid; EPA, eicosapentaenoic acid.

**Supplementary Table 4:** Plasma LM Profiles Post Albumin Administration

DHA bioactive metabolome	Q1	Q3	Group 1					Group 2				
			2-2529 AOB	3-2445 AOB	8-1822 AOB	9-2417 AOB	10-2200 AOB	5-1026 AOB	4-1486 AOB	1-2320 AOB	6-2419 AOB	7-1702 AOB
RvD1	375	141	0.3	—	0.4	2.1	1.1	—	2.5	9.4	0.4	3.4
RvD2	375	141	—	0.6	2.5	—	3.6	—	4.6	—	2.7	7.4
RvD3	375	147	—	0.7	—	6.7	—	3.4	3.1	0.8	0.4	—
RvD4	375	101	0.1	1.4	2.2	1.9	3.6	0.4	0.6	2.4	0.9	0.9
RvD5	359	199	3.6	3.6	5.6	14.1	18.2	7.4	7.8	1.6	7.5	17.5
RvD6	359	101	4.5	2.8	3.3	0.6	6.2	24.7	31.7	2.7	10.2	1.8
17R-RvD1	375	141	—	1.7	—	1.0	—	—	—	—	1.2	1.2
17R-RvD3	375	147	0.2	—	—	0.5	—	0.3	—	1.0	—	2.8
PD1	359	153	1.3	2.1	0.3	1.1	2.1	—	—	2.8	4.9	8.8
17R-PD1	359	153	5.1	3.9	—	1.5	3.4	—	—	—	—	1.5
10S,17S-diHDHA	359	153	—	—	—	—	0.9	—	—	—	0.6	—
22-OH-PD1	375	153	—	—	—	—	0.2	—	1.2	2.7	—	1.2
MaR1	359	221	—	—	—	—	1.7	2.9	—	—	—	—
7S,14S-diHDHA	359	221	3.1	—	1.9	20.0	3.2	—	—	8.6	3.2	—
4S,14S-diHDHA	359	101	—	—	1.6	6.6	—	—	—	19.5	—	3.8
n-3 DPA bioactive metabolome												
RvT1	377	239	0.5	0.5	0.6	0.5	0.5	7.4	1.7	0.7	0.8	8.8
RvT2	377	197	0.2	1.0	1.1	—	1.1	1.4	0.8	2.5	1.5	—
RvT3	377	197	0.6	0.3	0.6	—	0.8	—	0.6	3.8	0.3	2.7
RvT4	377	211	2.8	1.5	2.0	—	1.9	—	4.2	15.7	1.2	10.7
RvD1_n-3 DPA_	377	143	16.7	20.3	7.5	15.3	9.1	4.8	4.5	18.9	9.2	3.2
RvD2_n-3 DPA_	377	215	—	0.2	—	1.1	2.7	1.0	1.1	3.2	—	1.8
RvD5_n-3 DPA_	361	199	3.7	9.3	—	3.3	0.8	8.5	—	3.2	2.5	4.6
PD1_n-3 DPA_	361	183	24.4	47.3	299.5	108.5	41.5	574.0	422.6	—	—	367.1
10S,17S-diHDPA	361	183	—	—	—	—	—	—	—	—	—	—
MaR1_n-3 DPA_	361	223	—	4.7	10.3	78.1	2.7	—	13.0	—	—	109.7
7S, 14S-diHDPA	361	223	—	6.5	9.7	81.7	—	—	84.7	—	1.8	39.2
EPA bioactive metabolome												
RvE1	349	195	0.9	—	0.5	0.5	0.4	25.4	5.4	1.0	8.7	0.6
RvE2	333	199	3.9	—	1.9	1.2	1.0	207.1	39.1	4.3	32.3	2.5
RvE3	333	201	0.8	4.0	13.3	—	—	—	3.5	—	2.9	181.5
AA bioactive metabolome												
LXA_4_	351	217	0.7	—	—	0.4	—	1.5	0.4	—	1.5	0.7
LXB_4_	351	221	13.5	6.8	6.8	11.0	189.1	0.0	11.0	189.1	0.0	13.5
5S,15S-diHETE	335	235	58.8	182.7	47.9	71.2	21.4	282.4	126.7	58.6	11.0	45.2
AT-LXA_4_	351	217	—	—	—	3.5	—	17.6	3.5	—	17.6	—
AT-LXB_4_	351	221	—	—	—	—	—	129.3	—	—	129.3	—
LTB_4_	335	195	8.4	12.4	4.6	11.2	52.4	20.5	15.2	6.7	84.2	85.5
5S,12S-diHETE	335	195	13.2	4.3	2.0	11.2	52.4	5.7	10.3	8.1	56.8	4.2
20-OH-LTB_4_	351	195	15.6	4.2	—	—	—	19.5	5.2	26.5	129.3	—
LTC_4_	626	189	310.6	143.6	104.0	108.4	29.1	67.5	187.3	71.6	86.1	158.3
LTD_4_	497	189	—	—	181.7	—	—	—	—	—	11.8	—
LTE_4_	440	189	16.3	15.4	111.6	115.8	0.4	560.8	29.6	12.3	54.9	—
PGD_2_	351	189	21.2	34.8	26.8	13.9	47.3	36.9	13.5	14.5	27.4	32.8
PGE_2_	351	189	58.9	55.4	71.8	8.2	69.4	69.5	25.1	3.8	72.0	65.1
PGF_2α_	353	193	28.0	92.6	114.2	41.4	107.8	65.6	58.1	37.6	31.6	84.8
TxB_2_	369	169	3167.6	938.9	9291.8	943.0	4320.1	1790.9	3309.4	782.5	8298.5	5740.6

NOTE. LM levels were assessed using LM-metabololipidomics. Results are expressed as pg/mL. Em dash = below limit; limit ≈ 0.1 pg. With Q1, M-H (parent ion); and Q3, diagnostic ion in the tandem mass spectrometry (daughter ion).

AA, arachidonic acid; DHA, docosahexaenoic acid; DPA, docosapentaenoic acid; EPA, eicosapentaenoic acid; LM, lipid mediator; PG, prostaglandin.

**Supplementary Table 5:** Theoretical Calculations Comparing the Effects of Low and High Binding Affinity Albumin on Free Circulating Levels of PGE_2_ Based on Concentrations of PGE_2_ and Serum Albumin Found in Healthy Patients and Patients With ACLF

Albumin at 40 g/L (∼600 μM) (Healthy)					
PGE_2_, *pg/mL*	PGE_2_, *pM*	High binding affinity, *Kd μM*	Free PGE_2_, *pg/mL*	Low binding affinity, *Kd μM*	Free PGE_2_, *pg/mL*
2	5.7		0.00007		1.4
10	28.4	0.02	0.0003	200	2.5
20	56.7		0.0007		5

Albumin at 20 g/L (∼300 μM) (ACLF)					
PGE_2_, *pg/mL*	PGE_2_, *pM*	*Kd μM*	Free PGE_2_, *pg/mL*	*Kd μM*	Free PGE_2_, *pg/mL*
2	5.7		0.0001		0.80
10	28.4	0.02	0.0007	200	4.0
20	56.7		0.0013		7.99

ACLF, acute-on-chronic liver failure; PGE_2_, prostaglandin E_2_.

**Supplementary Table 6:** Plasma Cytokine and LPS Concentrations at Baseline and Following 20%-HAS Treatment Once Serum Albumin >30 g/L

	Mean plasma level pretreatment, *pg/mL* (n = 45)	Mean plasma level post-treatment, *pg/mL* ( n = 45)	Mean change post-treatment, *pg/mL*	CI, *pg/mL*
TNF-α	1.32	1.30	-0.01	-0.42 to 0.40
IL6	100.88	85.10	-17.46	-49.05 to 14.13
IL8	708.76	458.61	-252.80	-555.70 to 50.21
IL10	2.78	3.24	+0.45	-0.64 to 1.53
IL1β	1.28	1.14	-0.16	-0.99 to 0.68
LPS	17.71	15.69	-2.022	-4.792 to 0.7477

CI, confidence interval; HAS, human albumin solution; IL, interleukin; LPS, lipopolysaccharide; TNF, tumor necrosis factor.

## Supplementary Methods

### Plasma-Induced Monocyte-Derived Macrophage Dysfunction

Briefly, cultured human MDMs were stimulated with 1 ng/mL of LPS (*Salmonella abortus equi* S-form [TLR*grade*], Enzo Life Sciences) for 4 hours in the presence of 25% patient plasma pretreatment and post-treatment, and TNF production was measured (enzyme-linked immunosorbent assay, R&D Systems) as shown previously. Improvement in macrophage function was predefined as a significant increase in LPS-induced TNF production. Experiments were performed in the presence of plasma from healthy control subjects as a comparator. Pairs of patient plasma samples were identified that contained a pretreatment sample (serum albumin <30 g/L) and a subsequent post-treatment sample when serum albumin was first measured at >30 g/L.

## Plasma Protein Binding Capacity

To determine the amount of PGE_2_ bound, equilibrium dialysis using a Thermo Scientific Single-Use Rapid Equilibrium Dialysis Plate was used, which enabled quantification of bound versus free PGE_2_ via postdialysis sample scintillation counting. Patient plasma was incubated with H^3^-PGE_2_ mixed with unlabeled PGE_2_ (molar ratio, 2727:1; final concentration, 2.73 μM). This was then dialyzed against phosphate-buffered saline in the Rapid Equilibrium Dialysis Plate for 4 hours at 37°C. Counts in sample chamber and buffer chamber were measured enabling percentage or total concentration of PGE_2_ bound to be calculated (% bound = 100 – [cpm buffer chamber/cpm plasma chamber] × 100).

## Lipopolysaccharide Detection

HEK293 cells are transfected to stably express TLR4 and a nuclear factor-kB-inducible secreted embryonic alkaline phosphatase reporter gene. QUANTI-Blue detection medium changes color in the presence of secreted embryonic alkaline phosphatase in the spectrum of 620–655 nm. Because the absorbance is in direct proportion to the amount of endotoxin present, the concentration of endotoxin can be calculated from a standard curve obtained using serial dilutions of the HEK-Blue Endotoxin Standard (a preparation of *Escherichia coli* 055:B5 LPS standardized against Food and Drug Administration–approved control standard endotoxin). Samples were diluted in endotoxin-free water (Sigma, UK) and then incubated with the HEK293 cells for 24 hours. The supernatant from these cells was then incubated with the detection reagent for 4 hours before being read for absorbance at 640 nm on a FLUOStar Omega Plate reader (BMG Labtech, Ortenberg, Germany).

## Cytokine Measurement

Beads with the appropriate cytokines (IL1β, IL6, IL8, IL10, TNF-α) were mixed with standards as provided to produce a standard curve. Samples were diluted in sample diluent. Assay was then performed as per the instructions. Beads were read on a BD FACSVerse flow cytometer (3 lasers: 405 nm, 488 nm, and 640 nm; 10-parameter analysis; BD Biosciences). Data were acquired using BD FACSuite (BD Biosciences). Data were analyzed using FCAP Array software v3.0 (Soft Flow Inc, Hungary).

## Lipid Mediator Metabolomic Data

Plasma was placed in 4 volumes of ice cold methanol containing deuterium-labelled internal standards: d_4_-PGE_2_, d_5_-LXA_4_, d_5_-RvD2, d_4_-LTB_4_, d_5_-LTC_4_, d_5_-LTD_4_, d_5_-LTE_4_, and d_8_-5S-HETE (500 pg each; Cayman Chemicals). These were then kept at −20°C for 45 minutes to allow for protein precipitation and lipid mediators were extracted using C-18 based Solid Phase Extraction as in Colas et al^12^ (PubMed identifier: 24696140). Methyl formate fractions were brought to dryness using a TurboVap LP (Biotage) and products suspended in water-methanol (50:50 vol/vol) for liquid chromatography–tandem mass spectrometry based profiling. Here a Shimadzu LC-20AD HPLC and a Shimadzu SIL-20AC autoinjector (Shimadzu, Kyoto, Japan), paired with a QTrap 5500 (ABSciex, Warrington, UK) were used and operated as described in Colas et al^12^ (PubMed identifier: 24696140). To monitor each lipid mediator and deuterium-labelled internal standard, a multiple reaction monitoring method was developed using parent ions and characteristic diagnostic ion fragments as in Colas et al^12^ (PubMed identifier: 24696140). This was coupled to an information-dependent acquisition and an enhanced product ion scan. Identification criteria included matching retention time to synthetic standards and at least 6 diagnostic ions in the tandem mass spectrometry spectrum for each molecule. Calibration curves were obtained for each molecule using authentic and synthetic compound mixtures and deuterium-labelled lipid mediator at 0.78, 1.56, 3.12, 6.25, 12.5, 25, 50, 100, and 200 pg. Standards for liquid chromatography–tandem mass spectrometry profiling were produced biogenically, purchased from Cayman Chemicals, or provided by Dr Charles N. Serhan (supported by National Institutes of Health funded P01GM095467 to CNS). Linear calibration curves were obtained for each lipid mediator, which gave *r*^2^ values of 0.98–0.99.

## Trial Management and Monitoring

### Research Steering Group

The Research Steering Group operates on behalf of the funders to ensure that appropriate milestones have been met in the delivery of the trial. It consists of the chief investigator, an independent expert, and representatives of the Welcome Trust and Department of Health.

### Trial Management Group

The Trial Management Group comprises the chief investigator, clinical research fellow, clinical project manager, trial statistician, trial manager, data manager, health economist and P5 trial site principle investigator (PIs). The Trial Management Group is responsible for developing the design, coordination and strategic management of the trial.

### Trial Steering Committee

The Trial Steering Committee is the independent group responsible for oversight of the trial to safeguard the interests of trial patients. The Trial Steering Committee provides advice to the chief investigator, Clinical Trials Unit, funder, and sponsor on all aspects of the trial through its independent chair.

### Independent Data Monitoring Committee

The Independent Data Monitoring Committee is responsible for safeguarding the interests of trial patients, monitoring the accumulating data, and making recommendations to the Trial Steering Committee on whether the trial should continue as planned. It comprises a clinical chair (independent hepatologist), independent gastroenterologist, and an independent statistician all with expertise in clinical trials.

### Participating Hospitals and Personnel

Basildon University Hospital (Site PI Dr G. Wright), Bristol Royal Infirmary (Site PI Dr J. Portal), Singleton Hospital Swansea (Site PI Dr C. Lye Ch'ng), Newcastle Freeman Hospital (Site PI Dr S. McPherson), Royal London Hospital (Site PI Dr Y. Kallis), Hull Royal Infirmary (Site PI Dr Lynsey Corless), Royal Free Hospital (Site PI Professor R. Jalan), North Tees and Hartlepool NHS Foundation Trust (Site PI Professor Jane Metcalf), Royal Liverpool University Hospital (Site PI Dr P. Richardson), and University Hospitals Birmingham NHS Foundation Trust (Site PI Dr A. Elsharkawy).
